# From establishing a CHD-CMR unit to 3D printing in a developing country pediatric cardiac unit

**DOI:** 10.1186/1532-429X-18-S1-P179

**Published:** 2016-01-27

**Authors:** Mahesh Kappanayil, Rajesh Kannan

**Affiliations:** 1 grid.427788.6Pediatric Cardiology, Amrita Institute of Medical Sciences and Research Centre, Kochi, India; 2 grid.427788.6Radiology, Amrita Institute of Medical Sciences and Research Centre, Kochi, India

## Background

Units providing specialised, advanced care for children and adults with CHD are few and far-in-betwen in developing nations, despite having the greater proportion of the world's burden of CHD. CHD scores low among the health care priorities of the State in most low-medium income countries (LMIC).However, centers of excellence in CHD care have emerged even in these environments - with results matching those in developed nations. Among the last and least to develop in such limited resource environments are the modalities for advanced imaging. Cardiac MRI, in particular, has been late to emerge, and is yet to gain adequate foothold. Reasons : high infrastructural cost, highly expertise intensive, effor and time intensive. There are no dedicated CMR training programs in most developing countries including India. We attempt to trace the birth and steady growth of a dedicated CHD-CMR unit in a developing nation.

## Methods

Amrita Institute of Medical Sciences, Kochi, Kerala, India is one of the leading pediatric cardiac care units in the developing world. The pediatric cardiac program was established in 1998, and the unit does about 700 cardiac surgeries and an equal number of transcatheter interventions every year, with excellent results.

## Results

A dedicated CHD-CMR unit was established here in January 2010, with a 1.5Tesla GE magnet, one cardiac radiologist and one pediatric cardiologist. Neither of the personnel had recieved any formal training in CMR, except for short observerships and workshops overseas. Both cardiologist and radiologist continued to be involved in other spheres of their respective specialities, dedicating about 20% of their working hours to cardiac MRI. Much of their skill and expertise was gained over a period of time with real-world exposure to large number of patients with comolex CHD. Over 300 cardiac MRI studies were performed during this period, majority of them on complex unoperated/partially palliated complex CHD in patients older than 5 years age. Complex surgical procedures including biventricular repairs for complex structural lesions have been achieved with the use of CMR information. Finally, 3D printing of complex CHD using CMRI has enabled excellent surgical results in a difficult patient subset. Specific factors conducive to growth of CMR as a modality : large number of older children and young adults with complex CHD, who have not had corrective surgery, representing late survivors of CHD with complex hemodynamic interplay and adaptations.

## Conclusions

Cardiac MRI is ideal and best-suited for the developing nation mileu, provided the cost/expertise/infrastructural limitations can be overcome. Needs for the future : 1. Models for mentorship, with established Western units mentoring and training deveoping nation units. 2.Integration of such units into the global research strategies in cardiac MRI and its applications. 3.Vibrant exchange of knowledge between deveoped and developing nation CMRI units.

